# A Rapid Dual-Responsive Releasing Nano-Carrier by Decomposing the Copolymer and Reversing the Core Dissolution

**DOI:** 10.3389/fbioe.2021.784838

**Published:** 2021-11-04

**Authors:** Sen Liu, Can Shen, Cheng Qian, Jianquan Wang, Zhihao Wang, Xuecong Tang, Qiuyang Zhang, Changjiang Pan, Wei Ye

**Affiliations:** ^1^ Jiangsu Provincial Engineering Research Center for Biomedical Materials and Advanced Medical Devices, Faculty of Mechanical and Material Engineering, Huaiyin Institute of Technology, Huaian, China; ^2^ College of Engineering and Applied Sciences, Institute of Materials Engineering, Collaborative Innovation Center of Chemistry for Life Sciences, Nanjing University, Nanjing, China

**Keywords:** dual-responsive, nano-carrier, dissolution reversing, rapid drug release, tumor chemotherapy

## Abstract

The accumulation of nanotechnology-based drugs has been realized in various ways. However, the concentration of drugs encapsulated by nanomaterials is not equal to the concentration of effective drugs; often, the drugs become effective only when they are released from the nanomaterials as free drugs. This means only when the drugs are rapidly released after the accumulated drug-encapsulating nanomaterials can they truly achieve the purpose of increasing the concentration of drugs in the tumor. Therefore, we herein report a dual-response nano-carrier of glutathione and acid to achieve the rapid release of encapsulated drug and increase the effective drug concentration in the tumor. The nano-carrier was constructed using a dual-responsive amphiphilic copolymer, composed of polyethylene glycol and hydrophobic acetylated dextran and connected by a disulfide bond. In the tumor microenvironment, disulfide bonds could be biodegraded by glutathione that is overexpressed in the tumor, exposing the core of nano-carrier composed of acetylated dextran. Then the acidic environment would induce the deacetylation of acetylated dextran into water-soluble dextran. In this way, the nano-carrier will degrade quickly, realizing the purpose of rapid drug release. The results showed that the drug release rate of dual-responsive nano-carrier was much higher than that of glutathione or acid-responsive nano-carrier alone. Furthermore, both *in vitro* and *in vivo* experiments confirmed that dual-responsive nano-carrier possessed more efficient anti-tumor effects. Therefore, we believe that dual-responsive nano-carriers have better clinical application prospects.

## Introduction

Nano-carriers have played significant roles in improving the therapeutic effects of tumor chemotherapy in the past decades (Petros and Desimone, 2010; Shi et al., 2016). Therefore, currently, it is one of the most interesting research directions to explore more effective drug carriers to overcome tumors (Sun et al., 2017; Yao et al., 2020; Zhang et al., 2021). The rapid release of nano-carriers at the lesion site is an important property of ideal nano-carriers (Hou et al., 2019). Drugs exhibit their therapeutic activity only after being liberated from their carrier (Luck and Mason, 2013), and cancer cells have multiple mechanisms of multi-drug resistance to protect them from drugs, such as drug efflux (Ye et al., 2017; Liu et al., 2019), cell-intrinsic drug metabolism (Colombo et al., 1999), or detoxification (Wegiel et al., 2013). Thus, the drug release rate should be rapid enough to achieve a sufficiently high concentration in tumor cells, allowing the drugs to reach their targets and kill cancer cells.

Usually, the drugs are released from the carrier to become free drugs through the drug diffusion (Colombo et al., 1999) or carrier disintegration (Colombo et al., 1999; Yu et al., 2015a; Liu et al., 2019; Zhang et al., 2019; Cao et al., 2021) mechanisms. However, the rate of drug diffusion is relatively slow, usually with a half-life of several hours or days after release (Sun et al., 2012). Therefore, the carrier can be designed with the characteristics of rapid response dissociation so that they can quickly become free drugs at the lesion site to play their role in tumor inhibition.

**FIGURE 1:**
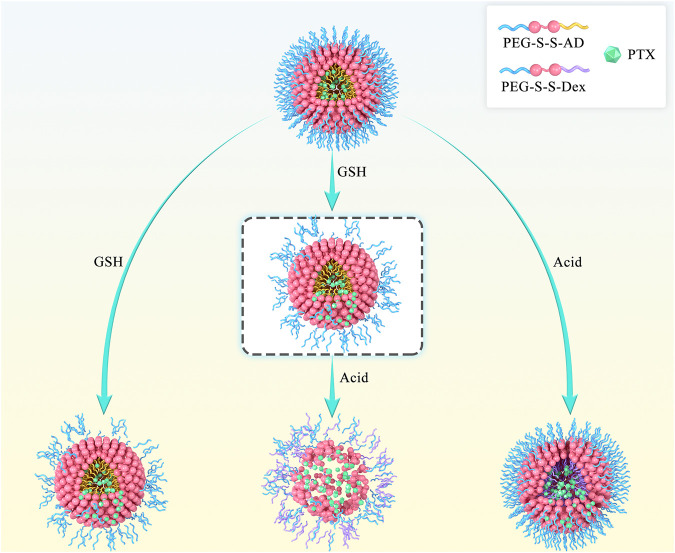
Scheme of rapid cleavage of dual-responsive nano-medicine (PTX@PSA NPs) in the presence of glutathione and acid. Compared to the shedding of the outer PEG layer induced by the breaking of disulfide bond in the presence of glutathione alone and the internal expansion induced by the change of hydrophobic AD to hydrophilic Dex in the presence of acid alone, dual-responsive PTX@PSA NPs possess a faster drug release rate.

Herein, we report a dual-responsive amphiphilic copolymer (PEG-SS-AD), composed of polyethylene glycol (PEG) and acetylated dextran (AD) linked by a disulfide bond (-S-S-). The rapid dual-responsive releasing nano-carrier (PSA NPs) was formed by the self-assembly of PEG-SS-AD. It is well-established that tumor cells overexpress glutathione (GSH) (Yan et al., 1991; Hou et al., 2019), which could act on -S-S- through biodegrading (Lappi and Ruddock, 2011; Hou et al., 2019). Therefore, the nano-carrier would degrade at the tumor site, and the water-soluble outer shell would be separated and dissolved in the -S-S- biodegrading process. The hydrophobic part was functionalized with acid-induced solubility reversible ability to accelerate the disintegration of the exposed hydrophobic core. Hydrophobic acetylated dextran (Dex) can be converted into hydrophilic Dex in the tumor’s acidic microenvironment (Dai et al., 2019). Hence, the dual-responsive nano-carrier is capable of rapid cleavage in the tumor (Figure 1). Once loaded with chemotherapeutic drugs, these nano-carriers can quickly release the coated drugs at the tumor site, increasing the concentration of the drug at the tumor site to enhance the effectiveness of chemotherapy.

## Experimental Section

### Materials

Methoxy-terminated polyethylene glycol (mPEG, Mw ≈ 2 KDa), carboxylic acid functionalized methoxyl polyethylene glycol (mPEG-COOH, Mw ≈ 2 KDa) were purchased from Ruixi Biological Technology Co., Ltd,. (Xi’an, China). N,N′-carbonyldiimidazole (CDI), 2,2-dithiodiethanol (DIT), succinic anhydride, dextran (Mw ≈ 2 KDa), 1-(3-Dimethylaminopropyl)-3-ethylcarbodiimide hydrochloride (EDC), N-Hydroxy succinimide (NHS), 2-methoxy propylene, pyridine p-toluene sulfonate, propionic anhydride, and dimethylaminopyridine (DMAP) were purchased from Energy Chemical (Shanghai, China). Water used in all the experiments was deionized (DI) water depurated by a Millipore ultrapure water system (Billerica, MA, United States) with a resistivity of 18.2 MΩ cm. High-glucose DMEM containing 1% penicillin/streptomycin, phosphate-buffered saline (PBS), and trypsin were obtained from KeyGen BioTech Co., Ltd,. (Jiangsu, China). Fetal bovine serum (FBS) was purchased from Absin Bioscience Inc (Shanghai, China).

### Synthesis of PEG-SS-AD (PSA)

Briefly, 4.0 g of mPEG and 0.162 g of CDI were dissolved in 50 ml of N,N-dimethyl formamide (DMF) and stirred for 24 h at room temperature. Then, 0.26 g of DIT was added and reacted at room temperature for another 12 h. The byproduct was then dialyzed and lyophilized to obtain disulfide bond-functionalized PEG (mPEG-SS, polymer 2).

Then, 2.2 g of mPEG-SS, dissolved in 50 ml DMF, was dissolved in 20 ml of DMF. Next, excess succinic anhydride was added and stirred at room temperature for 24 h. The byproduct was then dialyzed and lyophilized to obtain carboxyl-functionalized mPEG-SS (mPEG-SS-COOH, polymer 3).

In the next stage, 1.0 g of mPEG-SS-COOH and 1.0 g of Dex were dissolved in 20 ml of DMF, and 0.1 g of EDC and 0.06 g of NHS were added as catalysts. After 24 h of reaction, the byproduct was dialyzed and lyophilized to obtain PEG-SS-Dex (polymer 4).

Subsequently, 0.1 g of PEG-SS-Dex, 10 ml of 2-methoxy propylene, and 50 mg of pyridine p-toluene sulfonate were dissolved in 20 ml of dimethyl sulfoxide (DMSO). Then, the reactions were carried out at room temperature for 3 h. The byproduct was dialyzed and lyophilized to obtain PEG-SS-AD (polymer 5).

^1^H-NMR of the polymers was performed on a Bruker 400M (JNM-ECZS) with the CDCl_3_ solvent.

### Synthesis of PEG-propionic anhydride-modified dextran (PPD)

To synthesize PDP as a non-responsive control group, 1.2 g of mPEG-COOH, 1.0 g of Dex, and 97.5 mg of CDI were dissolved in 20 ml of DMSO, and the reactions were allowed to continue under stirring at 80°C overnight. Then, 5 ml of propionic anhydride, 100 mg of DMAP, and 1 ml of triethylamine were mixed and stirred at room temperature for 8 h. PPD was then obtained by dialysis and lyophilization.

### Preparation of Nano-Carriers (PSA NPs) and Nano-Medicine (PTX@PSA NPs)

PSA NPs were prepared by the self-assembly of PEG-SS-AD in the selective solvent of dichloromethane (DCM) in DI water, as reported previously (Hu et al., 2007; Yu et al., 2015b). Briefly, 10 mg of PEG-SS-AD was dissolved in 1 ml of DCM, and the mixture was slowly added to 10 ml of DI water. The mixture was then emulsified by ultra-sonication for 10 min. Finally, the emulsion was evaporated to remove DCM, followed by filtration and lyophilization. PTX@PSA NPs were obtained by adding 10 mg of PEG-SS-AD and 2 mg of PTX in 1 ml of DCM, and then the steps above were followed. Non-responsive PTX@PPD NPs were obtained by adding 10 mg of PEG-PD and 2 mg of PTX to 1 ml of DCM. Then the steps above were followed.

### Characterization of PSA NPs

To this end, 10 mg of PSA NPs was dissolved in 10 ml of alkalescent (pH ≈ 7.3) phosphate-buffered saline (PBS) solution, 0.2 μmol/L GSH alkalescent (pH ≈ 7.3) solution, acidic (pH ≈ 6.0) PBS solution, and 0.2 μmol/L GSH (pH ≈ 6.0) PBS solution, respectively, and incubated for 2 h. In addition, transmission electron microscopic (TEM, JEM-2100F) images were obtained to analyze changes in the morphology of PSA NPs under different conditions.

### Drug Loading Content and Encapsulation Efficiency

Standard solutions of PTX at concentrations of 0.001, 0.005, 0.01, 0.05, 0.1, 0.5, and 1 mg/ml were prepared to measure their ultraviolet absorption spectrum and evaluate the relationship between concentration and absorbance at 273 nm.

Then 10 mg of PTX@PSA NPs (W_PTX@PSA NPs_ = 10 mg) was dissolved in 4 ml of acetonitrile and PBS (pH = 7.4) (V/V = 2:3) mixed solvent and the supernatant was centrifuged. Then the concentration of PTX (C_PTX_) was measured by absorption photometry. The PTX loading content and encapsulation efficiency were calculated by (Eqs. 1, 2)

$$\mathrm{PTX loading content}=\frac{C_{PTX}\times 4\;mL}{W_{PTX@PSA\;NPs}}\times 100\%$$ (1)

$$\mathrm{PTX encapsulation efficiency}=\frac{C_{PTX}\times 4\;mL}{\mathrm{total amount of PTX}}\times 100\%$$ (2)

where the total amount of PTX = W_PTX@PSA NPs_/6 = 1.67 mg, according to the feeding ratio.

### *In Vitro* Controlled Release Ability

First, 5 ml of PTX@PSA NPs aqueous solutions (2 mg/ml) were sealed into dialysis tubes (MWCO 2 kDa) for four copies, and then immersed in 45 ml of alkalescent (pH ≈ 7.3) phosphate-buffered saline (PBS) solution, 0.2 μmol/L GSH alkalescent (pH ≈ 7.3) PBS solution, acidic (pH ≈ 6.0) PBS solution, and 0.2 μmol/L GSH acidic (pH ≈ 6.0) PBS solution, respectively. The drug release experiment was performed at 37°C, and three 1-ml solutions were taken from each group at 10 min and 0.5, 1, 3, 5, 8, 12, and 24 h, respectively. The concentration of PTX was measured by ultraviolet absorption spectrum, and the cumulative released PTX was calculated.

### *In vitro* cytotoxicity assay and anti-tumor effect

The cytotoxicity of 4T1 cells was tested using an MTT (3-(4,5-dimethylthiazol-2-yl)-2,5-diphenyltetrazolium bromide) assay. The cells were inoculated in 96-well plates, and when the cells covered approximately 80% of the total area, the medium was removed, and 100 μl of PSA NPs medium solution was added at concentrations of 0, 10, 10^2^, 10^3^, 10^4^, 10^5^, and 10^6^ ng/ml. After incubation for 24 h, the medium was removed, and the cells were washed three times with saline solution. Then 20 μl of MTT solution (2.5 mg ml−^1^ in saline solution) and 80 μl of culture medium were added per well, and the cells were incubated for another 4 h. Subsequently, the medium was aspirated, and 200 μl of DMSO solution was added to each well. After 15 min, the absorbance was measured at 490 nm using an iMark Enzyme mark instrument (Biotek Eon^TM^). The cell viability was calculated according to a previously reported approach.

The anti-tumor effects of PTX@PSA NPs and PTX@PPD NPs were tested using MTT assay by following the above method.

### *In vivo* Anti-Tumor Effects

A tumor-bearing mouse model was constructed by injecting 4T1 into the back of the mouse. When the tumor grew to approximately 100 mm^3^, they were divided into three groups (*n* = 10). Each mouse was intravenously injected with saline solution (100 μl) (group 1), PTX@PPD NPs (100 μl, 0.5 mg/ml) (group 2), and PTX@PSA NPs (100 μl, 0.5 mg/ml) (group 1). The treatment was performed twice a week for two weeks. Then the length (L, longest diameter) and width (W, shortest diameter) of the tumor were measured with vernier calipers every 3 days from the beginning of treatment, and the tumor volume was calculated using this Eq. (3):

$$V=\left(L\times W^{2}\right)/2$$ (3)

Three of the tumors in each group were harvested, and their optical pictures were obtained. The survival of the mice was determined every 3 days.

Three tumor-bearing mice were randomly selected and treated according to the above procedures. Tumors were harvested, isolated, immobilized, embedded into paraffin, cut into sections, stained with H&E, and observed under a microscope on the 14th day.

### Pathological Analysis

The tumor-bearing mice were intravenously injected with PSA NPs and PTX@PSA NPs, and then sacrificed on the 14th day. The main organs (heart, liver, spleen, lungs, kidneys) were harvested, isolated, fixed, embedded into paraffin, cut into sections, stained by H&E, and observed under a microscope.

## Results and Discussion

### Synthesis and Characterization of Dual-Responsive Amphiphilic Copolymers

**FIGURE 2:**
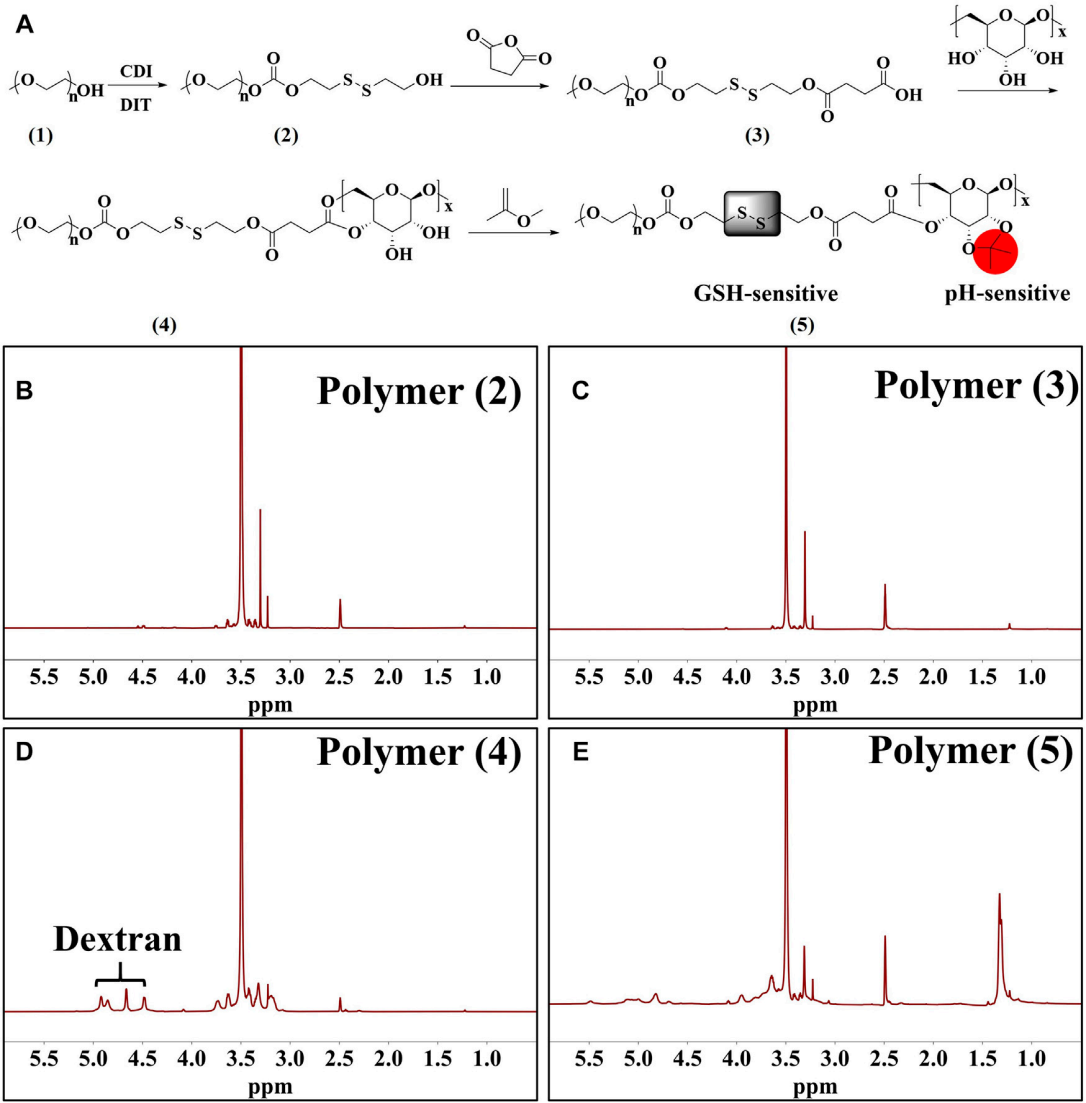
**(A)** Synthetic route of dual-responsive amphiphilic polymer; 1H NMR spectrum of polymers 2 to 5 **(B–E)**.

Figure 2A shows the synthesis pathway of GSH and pH dual-responsive PEG-SS-AD (Polymer 5). The GSH response was achieved by incorporating disulfide bonds between polyethylene glycol (PEG, Mw ≈ 2 KDa) and Dex (Mw ≈ 2 KDa) copolymer, while the pH response was achieved by acetylated dextran, which could be converted into hydrophilic dextran in the tumor’s acidic microenvironment. The products of polymers 2–5 were confirmed at each step by ^1^H NMR (Figures 2B–E). The absorption peak at 3.50 ppm represents the presence of PEG. The absorption peak of Dex at 4.48–4.92 ppm, which could be attributed to H atoms in -OH in each monomer of Dex, was detected in polymer 4, indicating that Dex was attached to PEG. In addition, after the acetylated dextran, the absorption peak of methyl (-CH_3_, the red part of polymer five in Figure 2A) appeared at 1.33 ppm, and the absorption peaks of -OH in Dex weakened, indicating the successful preparation of AD. These findings showed that the GSH and pH dual-responsive PEG-SS-AD were successfully synthesized according to the synthetic route.

### Characterization of Dual-Responsive Nano-Carriers

**FIGURE 3:**
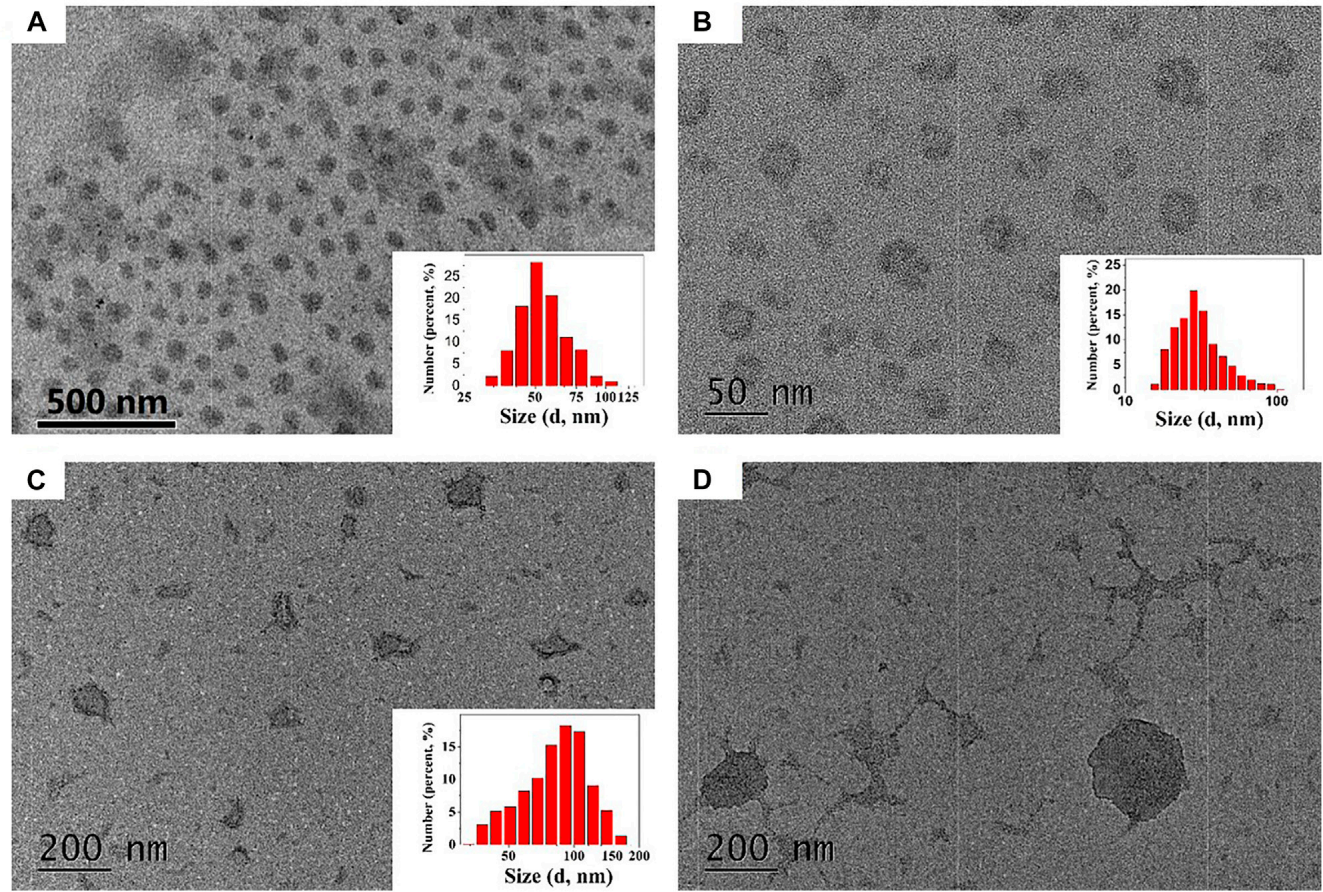
**(A)** The TEM images of PEG-SS-AD NPs (PSA NPs). **(B)** The TEM images of PEG-SS-AD NPs incubated in 0.2 μmol/L GSH alkalescent (pH ≈ 7.3) phosphate-buffered saline (PBS) solution. **(C)** The TEM images of PEG-SS-AD NPs incubated in acidic (pH ≈ 6.0) PBS solution. **(D)** The TEM images of PEG-SS-AD NPs incubated in 0.2 μmol/L GSH acidic (pH ≈ 6.0) PBS solution.

Then the nano-carriers were formed by the self-assembly of PEG-SS-AD in a selective solvent (PSA NPs), using the solvent evaporation method as we reported previously (Hu et al., 2007; Yu et al., 2015b). As shown in Figure 3A, PSA NPs have good water dispersion with relatively uniform size, i.e., approximately 40 nm. The mean hydrodynamic diameter of these PSA NPs was measured by dynamic light scattering (DLS), which was 55 nm with a polydispersity index (PdI) of 0.068 (inset). Then, PSA NPs were incubated in different solutions for 30 min, and their morphologies were observed under TEM, and mean hydrodynamic diameter was measured by DLS (Figure 3B–D). After incubating in 0.2 μmol/L GSH alkalescence (pH ≈ 7.3) phosphate-buffered saline (PBS) solution, the morphology of the PSA NPs was no longer regular, and some PSA NPs were worn out, and others were shrunk in size, with a mean hydrodynamic diameter of 41 nm and a higher PdI of 0.238 (Figure 3B). This might be due to the cleavage of -S-S- in PEG-SS-AD by GSH and the dissolution of the PEG shell. In addition, after incubating in acidic (pH ≈ 6.0) PBS solution without GSH, significant changes were observed in the morphology of PSA NPs, and more organic debris appeared, with a mean hydrodynamic diameter of 82.7 nm and a much higher PdI of 0.452 (Figure 3C). This might be attributed to the transition from hydrophobic AD to hydrophilic Dex in the acidic environment and the nano-carriers dissolved by the solvent. However, after incubating in 0.2 μmol/L GSH acidic (pH ≈ 6.0) PBS solution, there were no nano-carriers anymore, and all the nano-carriers degraded to organic fragments (Figure 3D). These findings indicated that PSA NPs possess the dual-responsive characteristics of GSH and acid and could disintegrate rapidly under the dual action of GSH and acid compared to GSH or acid alone. Encapsulation and controlled release properties of PTX@PSA NPs.

As PSA NPs possess dual-responsive characteristics of GSH and acid, PSA NPs encapsulating paclitaxel (PTX) (PTX@PSA NPs) were prepared, and the encapsulation and controlled release properties of PTX@PSA NPs were evaluated.

**FIGURE 4:**
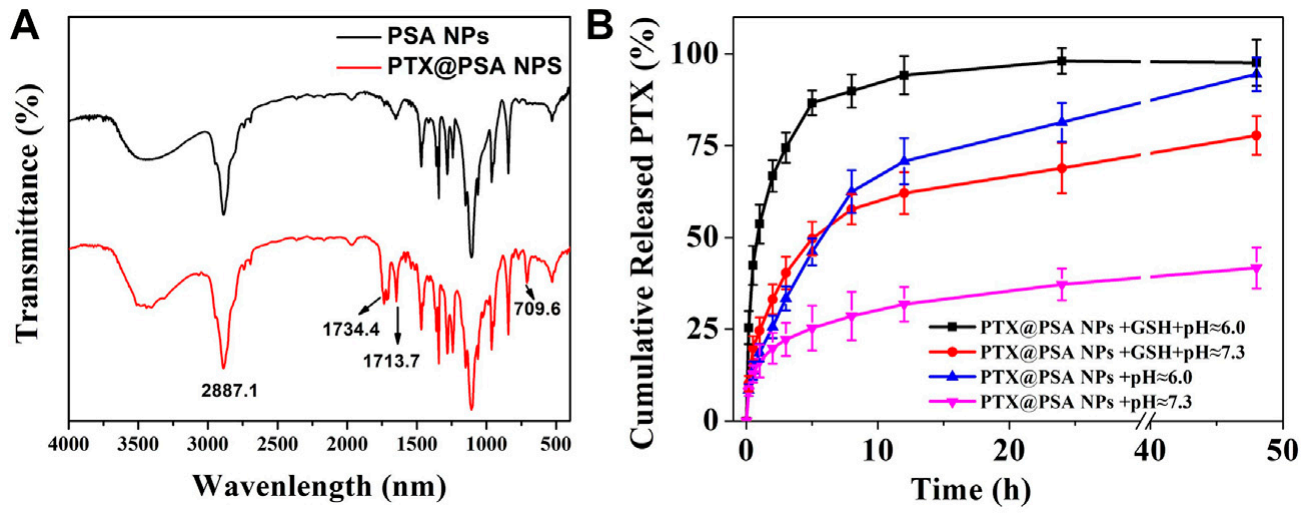
**(A)** FT-IR spectra of the nano-carriers (PSA NPs) and PTX encapsulation nano-medicine (PTX@PSA NPs). **(B)** *In vitro* release of CO from PTX@PSA NPs under stimulation without GSH and acid (PTX@PSA NPs + pH ≈ 7.3), acid (PTX@PSA NPs + pH ≈ 6.0), GSH (PTX@PSA NPs + GSH + pH ≈ 7.3), GSH and acid (PTX@PSA NPs + GSH + pH ≈ 6.0).

To prove the successful encapsulation of PTX by PSA NPs, the as-prepared PTX@PSA NPs were centrifuged and re-dispersed three times to fully remove the free PTX. FT-IR of PTX@PSA NPs and PSA NPs were then carried out, as shown in Figure 4A. The appearance of vibration peaks of the carbonyl group (1734.4, 1713.7 cm^−1^) and aromatic ring peak (709.6 cm^−1^) of PTX indicates that PTX was successfully encapsulated in PTX@PSA NPs. Through further testing, the PTX loading content and encapsulation efficiency of PTX@PSA NPs were calculated at 11.7 and 70.6%, according to Eqs. 1, 2 (Table 1).

**TABLE 1:** The PTX loading content and encapsulation efficiency of PTX@PSA NPs.

Weight of the PTX@PSA NPs (mg)	Concentration of PTX (μg/ml)	PTX loading content (wt%)	PTX encapsulation efficiency (wt%)
10	292.5	11.7	70.2

The cumulative *in vitro* release of PTX from PTX@PSA NPs was then measured under different conditions (Figure 4B). The release rate of PTX from PTX@PSA NPs was relatively slow in the absence of GSH and acid (pH ≈ 7.3); 19.8 ± 4.2% of the total amount was released in the first 2 h, most of which were probably the PTX adhering to the surface of PTX@PSA NPs, and only 41.7 ± 5.6% of the total amount was released at 48 h. Under the stimulation of GSH (GSH + pH ≈ 7.3) or acid (pH ≈ 6.0) alone, the PTX release rate increased, with 25.6 ± 3.1% and 33.1 ± 4.1% being released within 2 h, and 94.5 ± 4.6% and 77.8 ± 5.3% being released at 48 h, respectively. In contrast, under the combined action of GSH and acid (GSH + pH ≈ 6.0), the release rate of PTX increased significantly; 66.8 ± 4.3% of the total amount was released within 2 h, and almost all the drug was released after 12 h (94.2 ± 5.2%). The results were consistent with the morphologies of the nano-carriers under different conditions in Figure 3. The nano-carriers remained relatively stable under the weak alkaline condition without GSH, and the drug release rate was slow. Part of the nano-carriers degraded under the stimulation of GSH or acid, resulting in an increased PTX release rate. Under the dual stimulation of GSH and acid, the nano-carriers degraded rapidly, and the corresponding controlled release rate of drugs was very fast, releasing the coated PTX in a short time.

It is well-established that the tumor microenvironment is acidic, and overexpressed GSH, the dual-responsive nano-carriers designed here can rapidly degrade and release the encapsulated drugs into the tumor.

### *In vitro* Analysis

**FIGURE 5:**
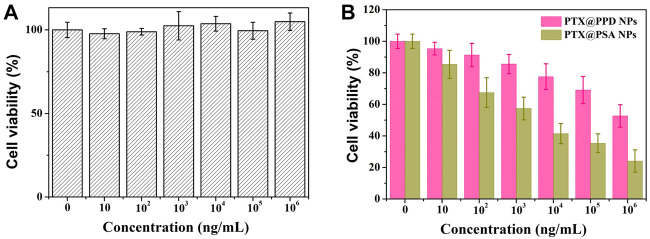
**(A)** Cytotoxicity profiles of 4T1 cells. **(B)** *In vitro* cell proliferation-inhibiting efficiency of PTX@PPD NPs and PTX@PSA NPs on 4T1 cells.

Although PEG and dextran have good biocompatibility, (Mura et al., 2013; Zhang et al., 2014) the safety of nano-carriers should be evaluated. The cytotoxicity of the PSA NPs was evaluated at different concentrations through the MTT assay using 4T1 cells. As shown in Figure 5A, PSA NPs showed no cytotoxicity. After co-culturing PSA NPs with 4T1 cells, cell proliferation was almost not inhibited under various concentrations of PSA NPs solution, and the cell’s survival rate was 104.9 ± 5.2% at a relatively high concentration of 1 mg/ml.

Then, propionic anhydride-modified dextran (PD), with no acid response characteristics, was successfully synthesized and directly connected to PEG without -S-S- to form a non-responsive amphiphilic copolymer (PEG-PD). Then non-responsive nano-medicine was prepared by PEG-PD encapsulating PTX (PTX@PPD NPs). Then the *in vitro* cell proliferation-inhibiting efficiency of PTX@PPD NPs and PTX@PSA NPs was evaluated. As shown in Figure 5B, both PTX@PPD NPs and PTX@PSA NPs showed cytotoxicity, but PTX@PSA NPs exhibited higher inhibitory efficiency on the proliferation of tumor cells. This might be because PTX@PSA NPs has a faster controlled release rate, forming a higher concentration of PTX, with a better tumor cell inhibition efficiency.

### *In vivo* Anti-Tumor Effects

Due to the EPR effect of solid tumors, nanoparticles measuring approximately 100 nm would accumulate in tumors (Lammers et al., 2012; Maeda et al., 2013; Wu, 2021). As the rapid, controlled release of PTX, PTX@PSA NPs showed could rapidly increase drug concentration and enhancing tumor cell inhibition *in vitro*. Thus, we speculated that PTX@PSA NPs would also show a significant anti-tumor effect *in vivo.* When the tumor size reached ∼100 mm^3^, tumor-bearing mice were equally divided into three groups (*n* = 10) and injected with normal saline solution (100 μl), PTX@PPD NPs, and PTX@PSA NPs, twice a week for 2 weeks.

**FIGURE 6:**
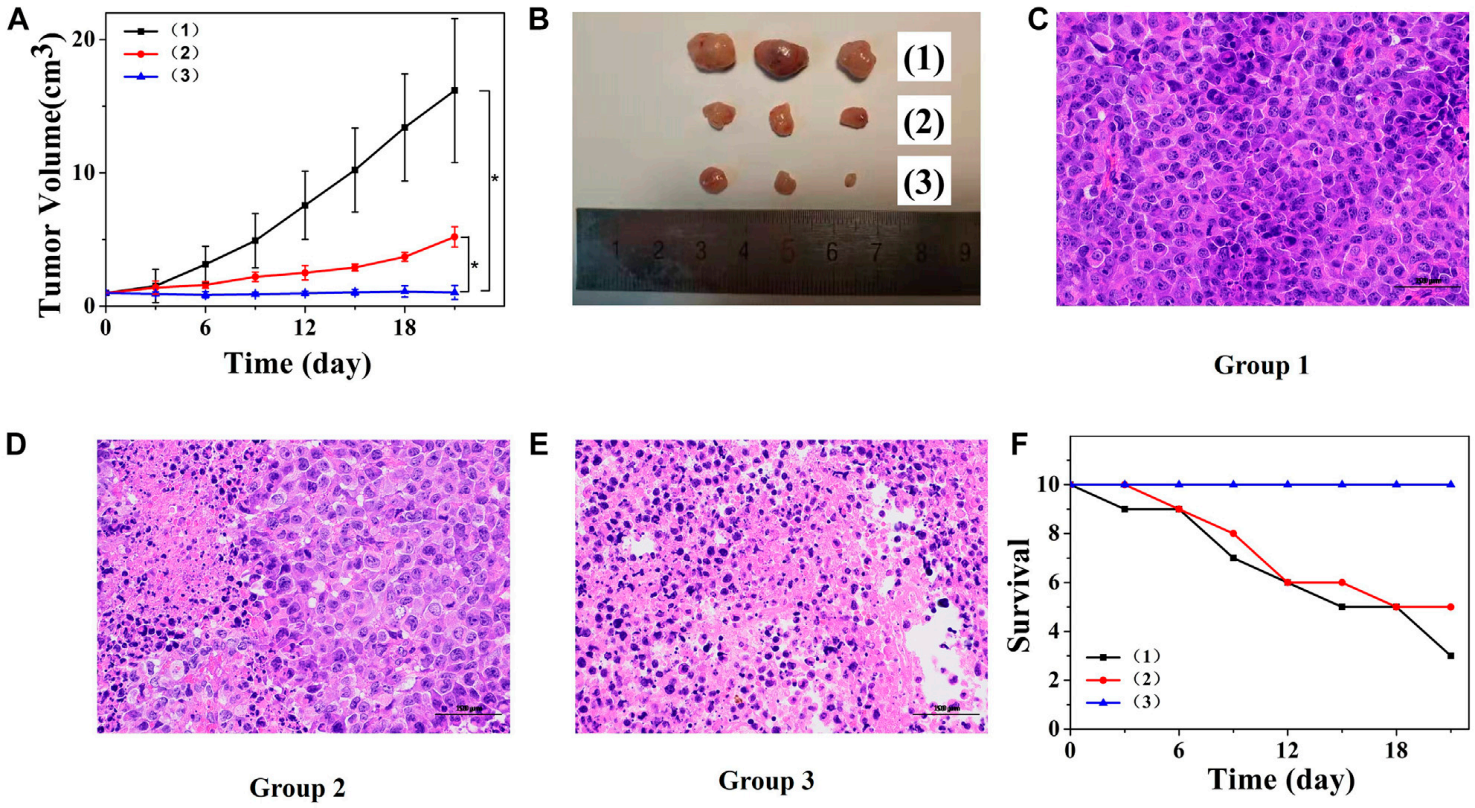
**(A)** Changes in tumor volumes of mice receiving different treatments. *p < 0.01. **(B)** Tumor images after treatment and observation period. **(C)** Pathological section of tumor tissue from group 1 via H&E staining. **(D)** Pathological section of tumor tissue from group 2 via H&E staining. **(E)** Pathological section of tumor tissue from group 3 via H&E staining. Scale bars: 100 μm. **(F)** Survival curves of mice in each group (*n* = 10 each group). Each mouse was intravenously injected with saline solution (100 μl) (1), PTX@PPD NPs (100 μl, 0.5 mg/ml) (2), and PTX@PSA NPs (100 μl, 0.5 mg/ml) (3).

The tumor volume was measured and calculated every 3 days, and the statistical results are presented in Figure 6A. The tumor volume in mice injected with saline solution increased rapidly. The tumor volume in mice injected with non-responsive PTX@PPD NPs was suppressed but still grew to 520 ± 76 mm^3^ on day 21. Dual-responsive PTX@PSA NPs showed promising tumor inhibition, and the tumor volume did not increase; it even decreased during the statistical period within 21 days. On day 21, optical camera images of three randomly selected tumors, presented in Figure 6B, showed the therapeutic effect. To further illustrate the effect of treatment on the tumor, three tumor-bearing mice were treated according to the above groups, and the tumoral sections of mice were harvested on the 14th day to observe the pathological changes (Figure 6C). The morphology of tumor cells in group 1 was almost unaffected. However, after treatment with non-responsive PTX@PPD NPs, the nuclei of some tumor cells were damaged, indicating the apoptosis of tumor cells (Figure 6D). However, after treatment with dual-responsive PTX@PSA NPs, tumor cells were significantly affected, and an extensive range of apoptosis appeared, indicating that dual-responsive PTX@PSA NPs have excellent *in vivo* tumor inhibition effect (Figure 6E).

Figure 6F presents the changes in the number of mice surviving after different treatments. Mice treated with saline solution died on day 3; seven of them died on day 21. However, the mice in the dual-responsive PTX@PSA NPs treatment did not die during the treatment and observation period, indicating that the dual-responsive PTX@PSA NPs can prolong the lifespan of mice by inhibiting tumor proliferation.

### Long-Term Pathological Study

**FIGURE 7:**
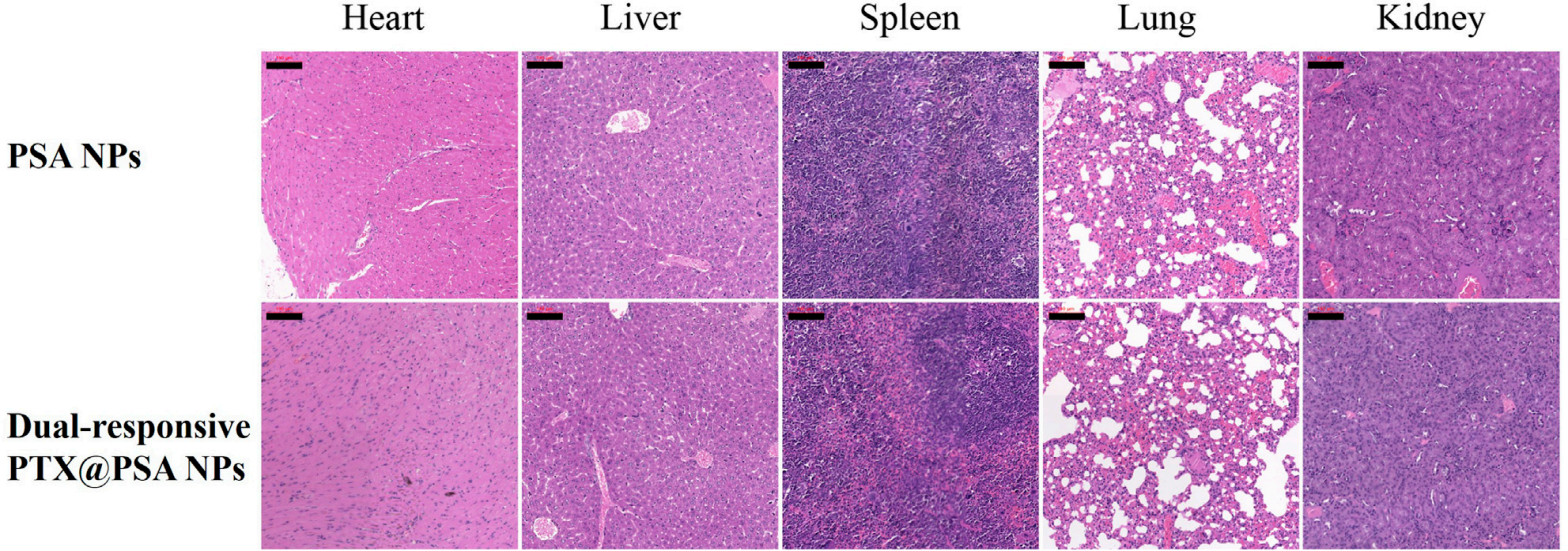
The cytotoxic effect caused by PSA NPs and PTX@PSA NPs in major organs via H&E staining (heart, liver, spleen, lungs, and kidneys). Scale bars: 100 μm.

The long-term *in vivo* safety of PSA NPs and PTX@PSA NPs were then further evaluated (Figure 7). Tumor-bearing mice received i.v., injections of 100-μl PSA NPs (0.5 mg/ml) and PTX@PSA NPs (0.5 mg/ml), respectively. Then, they were sacrificed on the 14th day after treatment, and their five major organs (heart, liver, spleen, lungs, and kidneys) were resected. H&E staining test was performed to evaluate the pathological condition. No apparent pathological changes, such as inflammatory lesions or abnormalities, were observed in the tissue sections of mice.

## Conclusion

In conclusion, we reported a dual-responsive nano-carrier constructed from an amphiphilic copolymer composed of -S-S- linked PEG and AD. The nano-carrier exhibited dual-responsive characteristics of GSH and acid and could quickly degrade and achieve rapid, controlled release of the drug under the interaction of GSH and acid, which was faster than the response of GSH or acid alone. Therefore, the dual-responsive PTX@PSA NPs possess a good tumor inhibition effect *in vivo* and *in vitro.* Furthermore, through the rapid, controlled release of PTX induced by the dual-responsive performance, the inhibitory effect was significantly better than that of non-responsive PTX@PPD NPs. Based on these conclusions, we believe that the rapid, controlled release of dual-responsive nano-carrier has more potential for clinical applications (Michael and Doherty, 2005).

## Data Availability

The original contributions presented in the study are included in the article/Supplementary Material, further inquiries can be directed to the corresponding authors.
